# Investigation of Needle Motion Profile Effect on Diesel Spray in Near-Nozzle Field

**DOI:** 10.3390/mi13111944

**Published:** 2022-11-10

**Authors:** Ya Gao, Weidi Huang, Raditya Hendra Pratama, Huifeng Gong, Jin Wang

**Affiliations:** 1 Advanced Photon Source, Argonne National Laboratory, 9700 S Cass Ave, Lemont, IL 60439, USA; 2 Research Institute for Energy Conservation, National Institute of Advanced Industrial Science and Technology, Namiki 1-2-1, Tsukuba 305-8564, Japan

**Keywords:** needle motion profiles, diesel spray, near-nozzle spray dynamics, x-ray imaging

## Abstract

A variety of needle-motion profiles are used in diesel injectors. However, it is unclear what the underlying mechanism is to determine the needle-motion profiles and how they affect the spray dynamics. It has been of significant interest to examine how the spray dynamics will change if only altering the needle valve opening speed or closing speed while all other parameters are kept the same. The different needle-motion profiles were obtained using a piezo nozzle (Nozzle #P) and a solenoid nozzle (Nozzle #S), which have identical nozzle geometry. By utilizing the X-ray imaging technique, it was observed that the average needle valve speed of Nozzle #P was 51% higher at the opening stage but 17% lower at the closing stage than Nozzle #S. When the needle valve lift is low (approximately 200 μm), the needle valve opening speed has a crucial effect on spray dynamics. The faster needle valve opening of Nozzle #P results in a 42% larger spray spreading angle and 34% lower spray velocity at the downstream field. The spray dynamics may be controllable by properly designing the needle-motion profiles in the scenarios of the low needle lifts. However, when the needle valve is sufficiently open (approximately over 200 μm), almost identical spray characteristics were observed regardless of the needle-motion profiles.

## 1. Introduction

Spray characteristics are known to have a predominated effect on diesel engine performance and emissions. Many factors can affect the diesel spray characteristics, one of which refers to the injector nozzle. The injector nozzle injects fuel at a pressure of hundreds of MPa and imparts the resulting spray with specific properties. Therefore, the geometry of the injector nozzle needs to be designed properly, mainly referring to the shapes of the nozzle hole, nozzle sac, and needle valve. Studies have been extensively conducted to reveal the effect of the hole diameter [1,2], hole number [3,4], hole shape [5,6], and sac shape [7,8] on the nozzle internal flow and spray characteristics. However, it is still challenging to quantify the link between the nozzle geometry and spray characteristics because the detailed understanding of the nozzle internal flow is insufficient yet. 

Another aspect regarding the injection nozzle effect on spray characteristics is the needle motion. In contrast to the geometry of the injector nozzle, the effect of needle motion on spray characteristics is more challenging to investigate. This is because, on the one hand, the transient dynamics must be considered when considering the needle motion effect. On the other hand, only limited techniques can measure the needle motion of the injector nozzle, mainly referring to the usage of optical nozzles [9,10] or optical sensors [11]. However, those methods are most suitable for low-pressure scenarios, which are different from the operating conditions of diesel nozzles. There have been some efforts to understand the needle motion effect in simulation [12,13,14,15,16]. However, experimental validation is vital for the simulation. In the early 2000s, X-ray diagnostics were introduced to examine the needle motion using a synchrotron X-ray source [17]. Later, by using the X-ray imaging technique, Powell et al. [18] examined the correlations between eccentric motions of the injector needle valve and oscillations in the fuel density. Moon et al. [19] investigated the emerging jet flows and related flow breakups at different needle lifts. Viera et al. [20] reported an experimental analysis of the relationship between instantaneous partial needle lifts and the corresponding injection rate. Most recently, Raditya et al. [21] discussed the needle lift dependency of the near-nozzle spray dynamics, mainly including the injection velocity and spray spreading angle at the nozzle exit. Bae et al. [22] proposed an understanding of the fuel-temperature effect on fuel injection performance by measuring the transient needle motion and injection velocity. 

Previous studies showed that the needle motion profiles are different in diesel nozzles. Two major categories of the needle motion profiles are commonly seen, including rectangle shape [18,20,23,24] and ramp shape [19,21,22,25,26]. It is also noted that the needle opening and closing speeds are varied among different nozzles. More specifically, some nozzles have almost the same needle valve opening and closing speed, but these speeds are different among the different nozzles, for instance, the results reported by [18] and [23]. As presented by [19,21], some nozzles have a higher needle valve opening speed relative to the closing speed, whereas some nozzles have the reverse trend, as seen in [22]. Despite a variety of needle motion profiles being used, it is unclear what the underlying mechanism is to determine the needle motion profiles and how they affect the spray dynamics. Therefore, it has been of significant interest to examine how the spray dynamics will change if only altering the needle valve rising speed or closing speed while all other parameters are kept the same. 

In this work, the X-ray imaging technique was used to investigate the needle profile (needle valve opening speed) effect on the spray dynamics in the near-nozzle field. The different needle motion profiles were obtained using a piezo nozzle and a solenoid nozzle, which have identical nozzle holes and sac geometry. By conducting this study, it is expected to contribute to a comprehensive understanding of the needle motion effect on spray dynamics. Nevertheless, the current investigation could result in following investigations both in experiment or simulation to discuss the effect of more sophisticated needle motion profiles on spray dynamics, for instance, two-steps-like.

## 2. Methods

### 2.1. Needle Motion Measurements

The needle motion measurements were carried out using the X-ray phase-contrast imaging (XPCI) technique at the 7IDB station of the Advanced Photron Source (APS). First, a brief introduction is made to the XPCI technique. When an X-ray beam propagates through an object, X-ray energy attenuates due to the energy absorption, and the phase shift arises simultaneously due to the diffraction. To record the needle motion in a thick steel housing, the XPCI technique is unique because the phase shift of X-ray between the edges of the needle valve and fuel can provide several orders of magnitude larger contrast compared to the energy attenuation. This feature ensures a sensitive detection of the needle valve edges, which benefits the analysis of needle motion afterward. Meanwhile, the high contrast from the XPCI technique can also increase the shutter speed to avoid motion blur using the high-speed camera. Compared to the usage of optical nozzles or optical sensors, the XPCI technique can directly image the needle motion in the operating conditions of diesel nozzles, i.e., at injection pressures over 200 MPa. In addition, the image resolution using XPCI can be as high as a sub-micrometer because synchrotron X-ray is highly collimated. These imaging specifications ensure tracing the needle motion during injection well.

Based on the time-sequential images of the needle motion captured by the high-speed camera, the time traces of the needle motion can be obtained by the cross-analysis of the needle images during the injection and before the injection. More specifically, a region of interest covering the needle tip (base window) was set in a frame before the needle opened, representing the initial needle location. Second, a searching window having the same size as the base window was set in the frame to calculate (regarded as the calculation frame). Next, the correlation coefficient *R* between these two windows could be obtained using the two-dimensional cross-correlation calculation, as Equation (1).

(1) $$R=\frac{\sum_{m}\sum_{n}\left(A_{mn}-\bar{A}\right)\left(B_{mn}-\bar{B}\right)}{\sqrt{\left(\sum_{m}\sum_{n}{\left(A_{mn}-\bar{A}\right)}^{2})\right)\sum_{m}\sum_{n}{\left(B_{mn}-\bar{B}\right)}^{2})}}$$

where *A* and *B* are the cropped images of the based window and the searching window, respectively. They have an image size of *m* × *n*, and $\bar{A}$ and $\bar{B\;}$ are the averaged gray value of *A* and *B*. By incrementally shifting the searching window’s location in the calculation frame and repeating the calculation using Equation (1), a series of *R* can be extracted corresponding to each searching window’s location. Their maximum indicates where the searching window’s cropped image perfectly matches the base window. Once the maximum of *R* is detected, needle lift can be known by counting the search window’s displacement relative to the base window. The details of the needle motion analysis technique can be found in our work [26,27]. 

### 2.2. Near-Nozzle Spray Dynamics 

**Figure 1:**
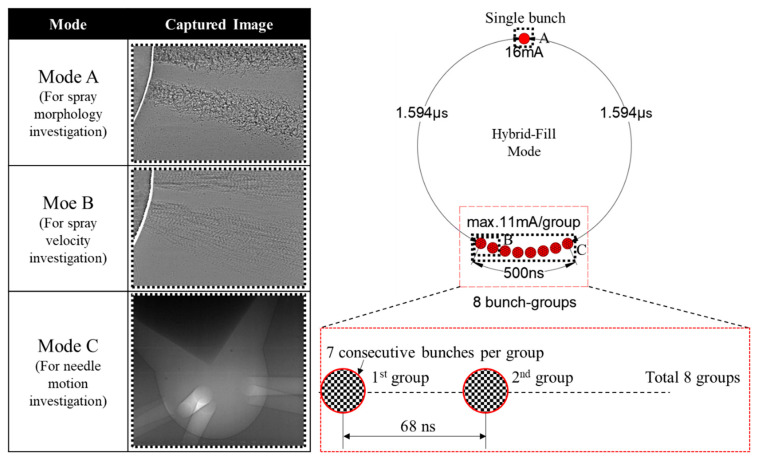
X-ray pulse timing patterns and modes used for single-exposure spray imaging, double-exposure spray imaging, and needle motion imaging.

The XPCI technique can also be used to measure the near-nozzle spray dynamics. There is an irregular beam mode at the APS, named the hybrid-fill mode. Figure 1 shows the pulse timing pattern of the hybrid-fill mode. It contains a single bunch with a 150 ps duration, and a 16 mA current isolated from the remaining 8 groups of 7 consecutive bunches (8 septets) by symmetrical 1.594 microseconds gaps. The group bunches have 11 mA current per group, a periodicity of 68 ns, with the total length of the bunch train being 500 ns. During the experiment, the single bunch, i.e., mode A was used for spray morphology investigation. This mode provided a limited pulse duration of 150 ps, which was beneficial for getting a still image of the spray.

A double-exposed spray image was obtained using two consecutive bunch groups, i.e., mode B in Figure 1. Based on an analysis of the double-exposed spray pattern on the image, the local velocity of the spray was known. More specifically, first, regions of interest (ROI) were selected at different locations along the spray axis of the double-exposed X-ray spray images. Then, an auto-correlation calculation was performed in the ROI at each location. The displacement vector of the imaged features during the 68 ns time interval can be obtained by detecting the relative location of the displacement peak concerning the center self-correlation peak. The details of the spray-velocity analysis technique can be found in our work [28,29]. 

Eight consecutive bunch groups, i.e., mode C, were used to get a bright image of needle motion inside the injector. To use different imaging modes, the pulse timing of the X-ray must be known. An imaging scan was done by shifting the camera shutter timing stepwise. Then, the pulse timing was known according to the intensity change in the sequence of images. Once the pulse timing was specified, different imaging purposes could be easily shifted using a digital delay generator. 

### 2.3. Experiment Setup 

**Figure 2:**
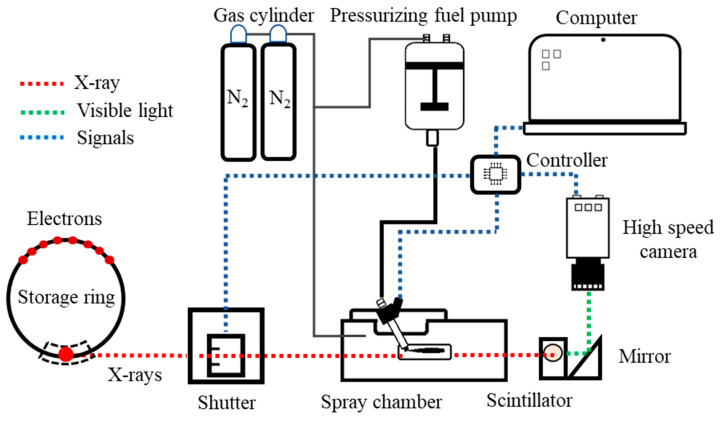
Schematic diagram of XPCI experiment.

The experiment setup is illustrated in Figure 2. Electrons circled in a storage ring under the guidance of insertion devices, i.e., different types of magnets. Whenever the electron-traveling path was changed, the electron emitted an X-ray beam along the tangent direction. The emitted X-ray beam was repositioned and refocused through a sophisticated setup of optic lenses and finally entered the experiment hutch for measuring uses. When the X-ray beam passed through the objective, a phase-contrast image was formed, which was converted using a scintillator crystal (LuAg:Ce) to visible lights. The synchrotron revolution frequency is 271 kHz. The higher the image rate, the smaller the image view field becomes. As a result, the high-speed camera used a reduced frequency signal as the triggering signal, i.e., once every four revolutions of the synchrotron. In addition, a mechanical chopper was placed upstream of the experiment system. This chopper enabled the X-ray to pass through only at the imaging instant. Otherwise, the scintillator crystal and camera could be damaged due to the heavy heat load of the X-ray. The converted visible-light image was recorded by the high-speed camera (Model SA-Z, Photron Inc., Tokyo, Japan). Several digital delay generators were used to synchronize the fuel injection and the timing of the incoming X-ray beam and the high-speed camera start-recording trigger. The view field of the images was 1.1 mm×1.1 mm, and the spatial resolution of the images was 2.5 μm/pixel. 

The details of the specification of the experimental apparatus are summarized in Table 1.

### 2.4. Experimental Conditions

The experimental conditions are presented in Table 2. Two diesel injectors were used in this investigation, which are a 7-hole piezo injector and a 7-hole solenoid injector. These two nozzles have the same hole inlet and outlet diameters, hole length, and sac volume. A common-rail diesel injection system powered by an air-driven mechanical pump delivered fuel to the injectors. The fuel used in this investigation was US Diesel #2. Three different injection-pulse widths were applied, including 0.3, 0.5, and 1.0 ms. The varying pulse widths provided a wide range of needle motion. The results of needle motion will be presented in the next section. The initial conditions in the spray chamber were fixed to the ambient pressure and room temperature using N_2_ as the ambient gas.

Regarding the experimental uncertainty, the current experiment mainly used the needle motion, spreading angle, and spray velocity for a quantitative discussion based on image analysis. The image resolution, i.e., 2.5 μm in the current investigation, is the primary factor of the resulting uncertainty. To minimize the resulting trend’s uncertainty, the ten-shot averaged results were used for the discussion, and the results’ standard deviation (STD) was also presented in the figures. The experiment was conducted at a fixed injection pressure of 200 MPa, which may fluctuate ±5 MPa due to the pump operating. According to a theory calculation, the fluctuation in injection pressure may result in a 1.2% injection velocity fluctuation.

## 3. Results

### 3.1. Needle Motion Results

**Figure 3:**
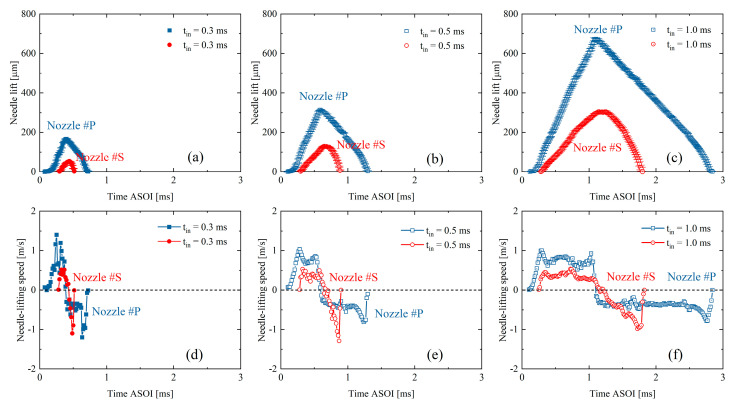
Results of needle motion: (**a**–**c**) needle lift, and (**d**–**f**) needle lifting speed.

Figure 3 shows the needle motion results of the testing nozzles. Time ASOI in the figure stands for the time after the injection triggering signal. Two testing nozzles both have the ramp shape of needle motion. The longer the injection pulse width, the higher the needle can rise. First, it can be seen that the needle motion is highly repeatable, mostly having an STD of less than two μm. The needle valve of Nozzle #P opens earlier compared to Nozzle #S. This result is understandable because piezoelectric materials generally respond faster than solenoid coils when they are energized. Once energized, the needle valve keeps lifting until the injection pulse is off. The maximum opening of the needle valve appears shortly after the set pulse-width time. The existing delay is because of the inertia of the needle valve opening. The average needle valve speed of Nozzle #P is 51 % higher at the opening stage but 17% lower at the closing stage compared to Nozzle #S. It is challenging to clarify the specific mechanism causing the different needle valve speeds in the nozzles because the details of the injector upstream part are unknown. The possible reasons could rely on the characteristics of piezoelectric materials, the springs’ stiffness, and other injector designs. However, the two testing nozzles having distinct needle motion profiles have been confirmed.

### 3.2. Spray Center Velocity at the Nozzle Exit

**Figure 4:**
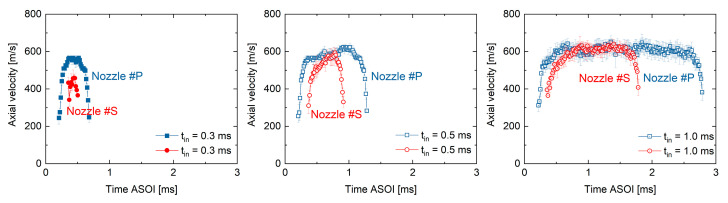
Time sequence results of spray axial velocities at the nozzle exit.

The effect of needle motion profiles on the near-nozzle spray dynamics will be examined in this section. First, Figure 4 shows the time-sequence results of spray center velocities, measured at 1.5 mm from the nozzle-hole exit. The time-sequence velocity profiles are approximately rectangular. The spray velocities in Nozzle #P appear earlier than in Nozzle #S. This is understandable due to its earlier needle valve opening, as shown in Figure 3. The velocity amplitude slightly increases with the extending injection pulse width. This phenomenon is particularly apparent in Nozzle #S. The nozzle-exit spray velocity is well known to primarily rely on the pressure differential across the nozzle hole [31,32]. Since the ambient gas pressure was kept constant in this investigation, the higher the sac pressure, the larger the spray axial velocity can reach. Therefore, the results in Figure 4 indicate that the sac pressure in Nozzle #S is still increasing when the injection pulse sets 0.3 ms and 0.5 ms. Once the fuel flow entering the sac and leaving from the nozzle holes reaches balance, the sac pressure will be stabilized, i.e., reaching a steady state. As a result, the spray velocity at the nozzle exit should also keep constant. The results in Figure 4 reveal that the sac pressure in Nozzle #P reaches a steady state earlier than in Nozzle #S. After reaching the steady state, the spray center velocities of Nozzle #P and Nozzle #S are almost identical.

**Figure 5:**
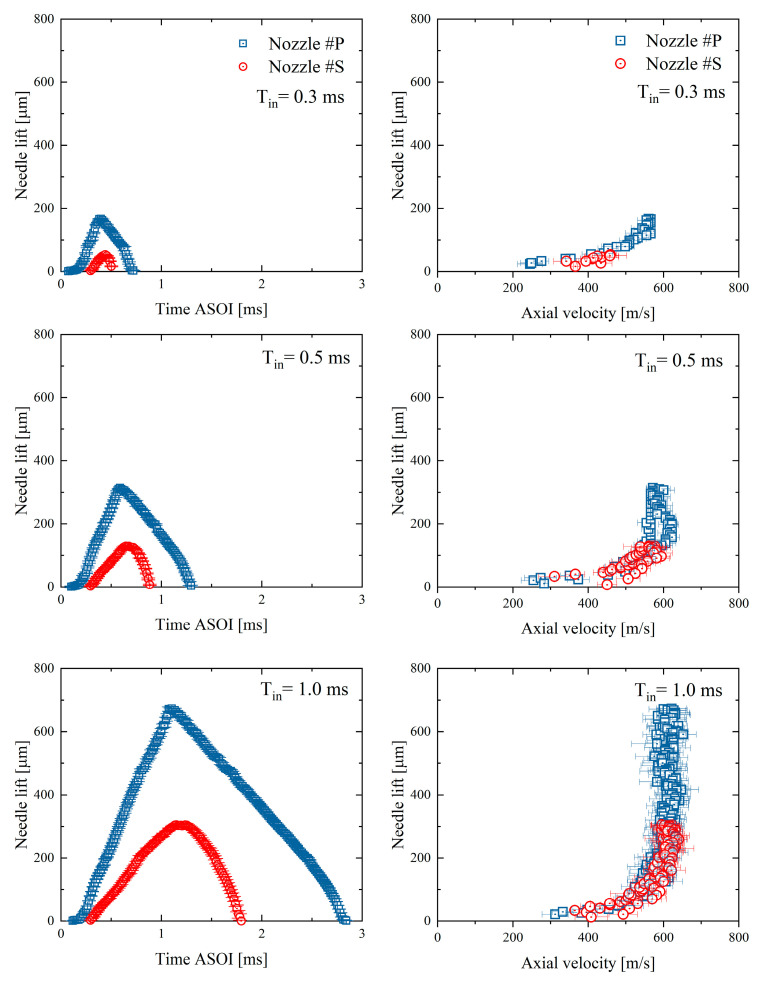
Needle lift sequence results of spray axial velocities at the nozzle exit.

Figure 5 presents spray center velocities in the needle lift sequence. The spray center velocities of Nozzle #P and Nozzle #S are almost overlapped in the needle lift sequence, indicating a significant dependency of the spray center velocity on needle lift regardless of the nozzle. When the needle is partially opened, the spray center velocity increases rapidly with the lifting needle valve. At the same needle lift, Nozzle #P and Nozzle #S have a similar amplitude of spray center velocity.

The spray can step into a steady state after a specific needle lift since when the spray center velocity will barely change. This critical needle lift was found to be approximately 200 μm for these two nozzles. It is worth noting that the critical needle lift will possibly change in the different nozzle geometry. More specifically, the needle valve shape and sac volume are the other important influencing factors of the building sac pressure. On the one hand, the needle valve shape can affect the flow rate entering the sac, which changes the building sac pressure.

On the other hand, the sac pressure also relies on the sac volume because the smaller the sac volume, the faster the sac pressure can rise when the identical fuel mass enters the sac. It might also be noted that the spray velocities during the opening stage and closing stages are slightly varied even at the same needle lift. This phenomenon is likely explained as the dynamic flow lag or flow hysteresis [33,34]. In this investigation, the needle motion effect on the spray dynamics was discussed, focusing on the needle opening stage.

### 3.3. Spray Spreading Angle at the Nozzle Exit

**Figure 6:**
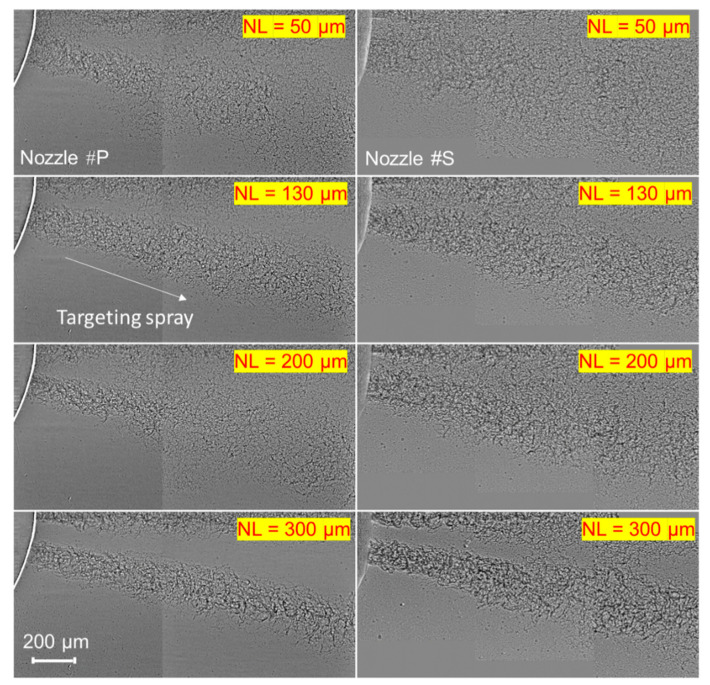
High-speed images of the injected sprays at the same needle lifts. (*NL* stands for the needle lift, and the targeting spray is inclined).

This section will discuss the spray spreading angle at the nozzle exit. First, Figure 6 shows spray morphology within 2 mm from the nozzle exit. The images of Nozzle #P and Nozzle #S captured at the same needle lifts were compared. At a needle lift of 50 µm, the sprays of both nozzles disperse widely, and the gap between two adjacent sprays almost disappears. A previous investigation [35] reported that the fuel flow would flush into the sac first rather than directly entering the nozzle hole under an extremely low needle lift. A vortex could form in this period, resulting in significant flow turbulence. Once this high-degree turbulent flow leaves the nozzle hole, it spreads immediately and widely. The current experimental results can be understood with the explanation above. The sprays get slimmer when the needle lift arrives at 130 µm and later at 200 µm. The spray of Nozzle #S seems to spread even less compared to Nozzle #P. When the needle lift reaches 300 µm, the sprays shrink obviously in both nozzles.

**Figure 7:**
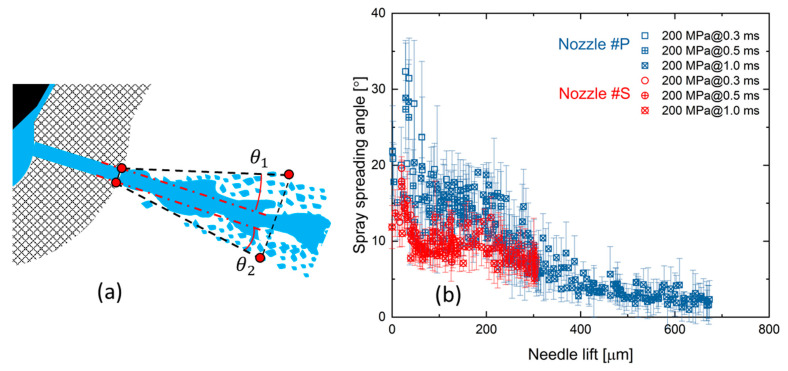
Comparison of the spray spreading angle between two nozzles: (**a**) the definition of spray spreading angle; (**b**) the results of spray spreading angle.

The spray spreading angle was further measured quantitatively based on a rectangle drawn from the nozzle exit until 1.5 mm, as shown in Figure 7a. More specifically, imaging processing codes were created to automatically detect the spray boundary at 1.5 mm from the nozzle exit. Then, the spray spreading angle was defined as the sum of the rectangle’s upper ($\theta_{1}$) and bottom parts angles ($\theta_{2}$). There were some cases (mostly when the needle lift was less than 50 µm) where the spray spread so much that $\theta_{1}$ was not recognizable. In such cases, the spray spreading angle was defined as twice $\theta_{2}$ instead. This method should be reasonable because the large spreading angle is mainly due to the high-degree flow turbulence. The spray dynamics tend to distribute uniformly when the flow turbulence is high. It deserves mentioning that the conventional spray angles are measured in the downstream spray field (typically 50 mm from the nozzle exit or farther), where the aerodynamic effect has engaged in spray characteristics. In contrast, the spreading angles were derived adjacent to the nozzle exit, which should be more closely related to the nozzle’s internal flow characteristics. As a result, the spreading angle and its STD are used as indicators of the internal flow turbulence.

Figure 7b shows the results of the spray spreading angle. It is seen that the spreading angle of Nozzle #P is 42% larger than Nozzle #S within a needle lift ranging from 0 µm to 200 µm, approximately. When the needle lift exceeds 200 µm, the spreading angles of the two nozzles become identical. As mentioned above, the spray spreading angle mainly depends on the internal flow because the current spreading angle was measured at 1.5 mm from the nozzle exit. At such a distance, the aerodynamics should not have been effective yet. Therefore, the results shown in Figure 7b reveal that a higher degree of flow turbulence exists in Nozzle #P compared to Nozzle #S at the low needle lifts. However, the flow turbulence in both nozzles will become identical at the high needle lifts.

### 3.4. Spray Dynamic Evolution in the Near-Nozzle Field

**Figure 8:**
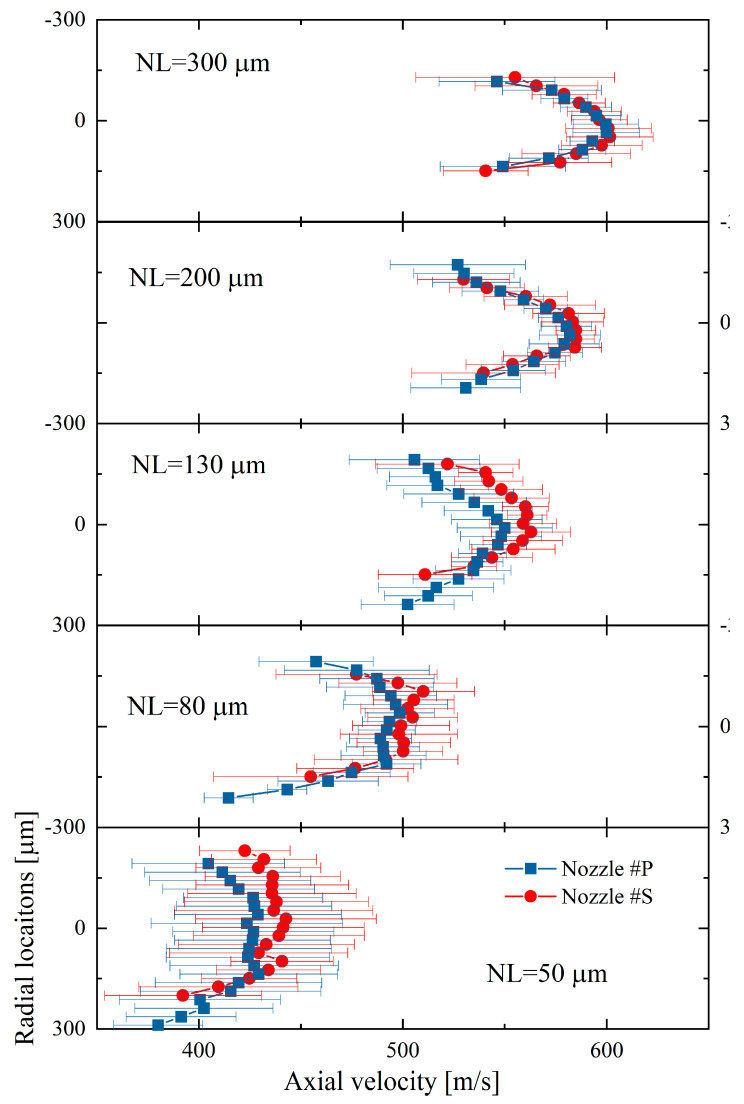
Nozzle−exit spray velocity distributions at different needle lifts.

The spray dynamic evolution in the near-nozzle field is further examined in this section. First, Figure 8 compares the spray velocity distributions in radial locations. The velocity distribution results were obtained at 1.5 mm from the nozzle exit under an injection pulse width of 1.0 ms. The velocity distribution at 1.5 mm from the nozzle exit is considered to represent the internal flow characteristics.

It is seen that the spray velocity distribution changes with the increasing needle lift. At the low needle lifts, i.e., 50 and 80 µm, the spray velocities are almost identical in the central region, whereas they decline rapidly in the periphery region. This distribution is typically called a top-hat shape of velocity distribution. The velocity distribution changes gradually from a top-hat shape to a parabolic shape with the lifting needle valve. This phenomenon is likely explained because the higher the flow turbulence, the more uniform the spray velocity distributes. The flow turbulence can also be known from the highly dispersed spray spreading angle. The spray becomes less turbulent at the high needle lift (200 and 300 μm), and the parabolic velocity distribution appears. Regarding the differences in the velocity distribution between the two nozzles, Nozzle #P has an overall lower velocity and flatter velocity distribution than Nozzle #S when the needle lifts are 50, 80, and 130 μm. However, as the needle lift increases, the velocity distribution of Nozzle #P and Nozzle #S becomes almost identical at the needle lift of 200 and 300 μm.

**Figure 9:**
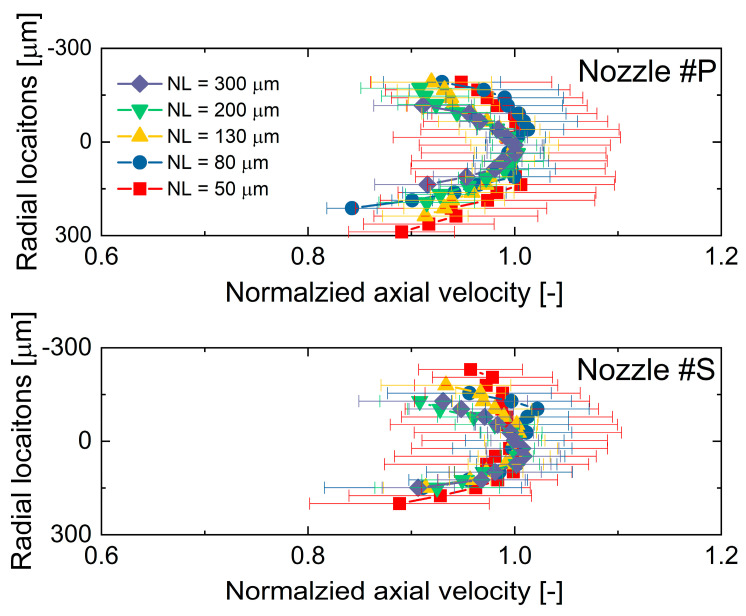
Normalized nozzle−exit spray velocity distributions at different needle lifts.

In Figure 8, it is seen that the spray velocity gradually increases with the lifting needle valve. To compare the velocity distribution at different needle lifts, the spray velocities ($U_{r}$) were normalized by the spray center velocity ($U_{r=0}$) at each needle lift, as shown in Equation (2).

(2) $$\bar{U_{r}}=U_{r}/U_{r=0}$$

where *r* is the radial location. The normalized nozzle-exit spray velocity distributions at different needle lifts are presented in Figure 9. It is evident that nozzle #S has a slightly narrower velocity distribution compared to Nozzle #P, especially at the low needle lifts. This result matches well with the observation in the spray spreading angle that the spreading angle of Nozzle #S is smaller than Nozzle #P when the needle lift is low. With the needle lift, the velocity distribution changes from top-hat to parabolic. At the 200 and 300 µm needle lifts, the velocity distributions are almost overlapped regardless of nozzle motion. It might also be noted that the fluctuation of the spray velocity, i.e., the error bar of spray velocity (ten shots were measured at each condition), is relatively larger than the results of needle motion. This is because the spray velocity, as well as the spray spreading angle, are also affected by flow turbulent instability. The STD of spray velocity decreases significantly with the lifting needle valve, indicating that the flow turbulence reduces at the high needle lifts.

**Figure 10:**
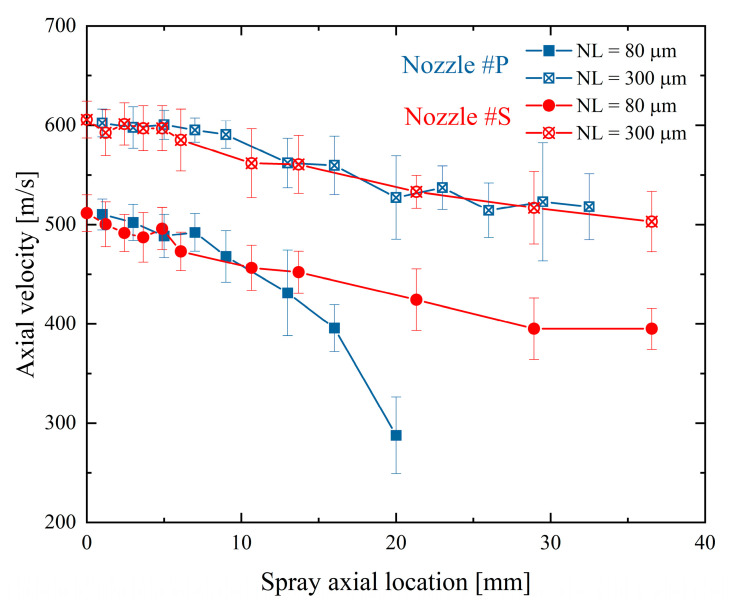
Spray velocity evolutions along the spray axis at the two different needle lifts.

Figure 10 shows spray velocity evolutions along the spray axis. The results of the needle lifts of 80 and 300 µm under an injection pulse width of 1.0 ms were illustrated only as an example. The spray velocity of Nozzle #P declines rapidly along the spray axis at the 80 µm needle lift. In contrast, the spray velocities along the spray axis of Nozzle #P and Nozzle #S have become identical at the needle lift of 300 µm. This result is consistent with the results in the previous sections, showing that Nozzle #P and Nozzle #S have similar spray center velocities and spray spreading angles at the high needle lifts. Therefore, it is understandable that these two nozzles have an identical velocity distribution along the spray axis. It is worth noting that the spray velocity of Nozzle #S increases with the increasing needle lift, whereas the velocity declination rate along the spray axis is almost unchanged. Therefore, it is considered that Nozzle #S should have less flow turbulence inside the nozzle regardless of the needle lift because, as shown in Figure 7b, the spray spreading angles of Nozzle #S are similar at the needle lift of 80 µm and 300 µm. As such, the change in spray velocity is mainly related to the pressure increase in the nozzle sac.

## 4. Discussion

**Figure 11:**
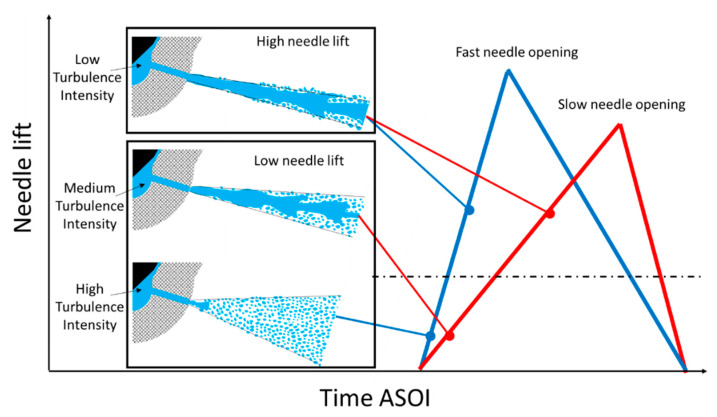
Needle motion profile effect on the spray dynamics.

The spray dynamics of Nozzle #P and Nozzle #S can be summarized as shown in Figure 11. First, a faster needle-valve opening of Nozzle #P results in a larger spray spreading angle than Nozzle #S, even if the needle lift is the same. The larger spray spreading angle and its STD results likely indicate a higher degree of flow turbulence. As a result, faster needle valve opening was observed as well to have a slightly lower spray center velocity at the nozzle exit and a fast velocity decay along the spray axis. The injection pulse is increasingly shortened in modern diesel engines to achieve ultra-optimized combustion control. In such scenarios, the needle lifts are typically low, where the spray dynamics can be controlled by changing the needle valve opening speed according to the current investigation.

With the needle valve further opening, a critical needle lift exists (200 μm in the current investigation) when the spray dynamics turn into a steady state. This change should relate to stabilizing sac pressure and nozzle internal flow. Two pieces of evidence are found to support this argument. One is that the spray spreading angle becomes smaller, and the other is that the STD of the spray spreading angle and spray velocity are largely reduced compared to the low needle lift. When the needle valve exceeds the critical lift, the spray dynamics will keep constant, i.e., being insensitive to the needle valve opening speed and the height of the needle lift. Almost identical spray characteristics were observed regardless of needle motion profiles, including the spray center velocities, spreading angles at the nozzle exit, and the spray dynamic evolution in the near-nozzle field.

Nozzle #P and Nozzle #S have identical nozzle holes and sac geometry but different needle motion profiles. More specifically, Nozzle #P has a faster needle opening and slower needle closing compared to Nozzle #S. Regarding the mechanism of why Nozzle #P, i.e., the faster needle valve opening tends to cause a larger spray spreading angle and faster velocity decay along the spray axis at the low needle lifts, a detailed simulation might be needed in the future. However, as a simplified analysis, the near-nozzle spray dynamics are typically regarded to rely on the nozzle internal flow characteristics. Second, the internal flow turbulence mainly relates to the flow momentum inside the nozzle sac, which further links to the time gradient of the sac pressure change [26,35]. Then, the time gradient of sac pressure change can be correlated with the needle lift using Equation (3),

(3) $$\frac{dp}{dt}=\frac{dp/dl}{dt/dl}=V_{NL}\times \frac{dp}{dl}$$

where p and l are the sac pressure and needle lift, respectively. $V_{NL}\;$stands for the needle valve opening speed. It can be known from Equation (2) that the faster the needle valve opens, the larger the time gradient of the sac pressure change is. Therefore, it is reasonable that a faster needle opening can result in more flow turbulence. On the other hand, the spray velocity also depends on the absolute sac pressure. Once the sac pressure reaches a balance, i.e., being identical to the common rail pressure, the time gradient of the sac pressure will not change anymore. Therefore, the flow turbulence in the nozzle significantly reduces, making the spray dynamics irrelevant to the needle valve opening speed and the height of the needle lift.

## 5. Conclusions

A variety of needle motion profiles are extensively used in diesel injectors. However, it is unclear what the underlying mechanism is to determine the needle motion profiles and how they affect the spray dynamics. In this work, the effect of the needle motion profiles on diesel spray in the near-nozzle field was investigated. First, two needle motion profiles were obtained using a piezo nozzle (Nozzle #P) and a solenoid nozzle (Nozzle #S), which have almost identical nozzle holes and sac geometry. Then, by utilizing the X-ray imaging technique, the needle motion, spray center velocities, spreading angles at the nozzle exit, and the spray dynamic evolution in the near-nozzle field were quantitatively measured. The results were further discussed in detail. The key findings of this study can be summarized below.

1. Nozzle #P has a shorter opening delay compared to Nozzle #S. This result should rely on the fact that piezoelectric materials generally respond faster than solenoid coils when they are energized. The average needle valve speed of Nozzle #P was 51% higher at the opening stage but 17% lower than Nozzle #S at the closing stage.
2. When the needle valve lift is low (approximately 200 μm in the current investigation), the needle valve opening speed has a crucial effect on spray dynamics. The faster needle valve opening of Nozzle #P results in a 42% larger spray spreading angle and 34% lower spray velocity at the downstream field. These results reveal significant differences in the in-nozzle flow characteristics due to differences in needle valve opening speeds. When the needle valve is sufficiently open (approximately over 200 μm), almost identical spray characteristics were observed regardless of the needle motion profiles.
3. According to a simplified analytical analysis, the faster needle opening speed can result in a larger time gradient of sac pressure change, a more rapid change in the flow energy inside the nozzle, and a higher degree of flow turbulence. However, as the stabilizing sac pressure, the flow turbulence in the nozzle greatly reduces. Therefore, the spray dynamics become insensitive to the needle motion profiles at the high needle lifts.
4. The needle motion profile effect is found to be particularly evident at the low needle lifts. The injection pulse is increasingly shortened in modern diesel engines to achieve ultra-optimized combustion control. In such scenarios, the needle lifts are typically low, where the spray dynamics can be controlled by adequately designing the needle motion profiles.

## Figures and Tables

**Table 1:** Specification of the experimental apparatus.

Experimental Apparatus	Specification	Remark
Experimental X-rays	at APS 7IDB beamline	Special Operating Mode—hybrid fill, top-up [30]
Pressurizing fuel pump	HII 5L-DD-300	Maximum Outlet Pressure 3103 bar
Spray chamber window	Kapton film of 12 × 30 mm	Maximum Ambient Pressure11 bar
High-speed camera	FASTCAM SA-Z	1024 × 1000 pixels at 21000 fps
Scintillator	LuAG:Ce Crystals	10 mm Diameter 0.1 mm Thickness
Controller	Digital Delay Generator(DG 535)	Delay resolution of 5 psChannel Jitter of 50 ps

**Table 2:** Experimental conditions.

**Nozzle Geometries**		
Injectors	Nozzle #P	Nozzle #S
Specification	7-hole piezo injector	7-hole solenoid injector
Hole diameters	Inlet: 0.14 mm	
	Outlet: 0.12 mm	
Hole length	0.80 mm	
Sac volume	0.22 mm^3^	
**Initial conditions**		
Fuels	Us Diesel #2	
Injection pressure	200 MPa (±5 MPa)	
Injection pulse widths	0.3, 0.5, 1.0 ms	
Ambient conditions	N_2_ at 1.25 kg/m^3^ and 25 ℃	
Measuring repetitions	Ten shots	

## Data Availability

Not applicable.
